# Associations Between Vaginal Bacteria and Bacterial Vaginosis Signs and Symptoms: A Comparative Study of Kenyan and American Women

**DOI:** 10.3389/fcimb.2022.801770

**Published:** 2022-03-04

**Authors:** Kayla A. Carter, Jennifer E. Balkus, Omu Anzala, Joshua Kimani, Noah G. Hoffman, Tina L. Fiedler, Vernon Mochache, David N. Fredricks, Raymond Scott McClelland, Sujatha Srinivasan

**Affiliations:** ^1^ Department of Epidemiology, University of Washington, Seattle, WA, United States; ^2^ Vaccine and Infectious Disease Division, Fred Hutchinson Cancer Research Center, Seattle, WA, United States; ^3^ Department of Medical Microbiology, University of Nairobi, Nairobi, Kenya; ^4^ Institute for Tropical and Infectious Diseases, University of Nairobi, Nairobi, Kenya; ^5^ Department of Laboratory Medicine and Pathology, University of Washington, Seattle, WA, United States; ^6^ Centre for Public Health Research, Kenya Medical Research Institute, Nairobi, Kenya; ^7^ Department of Medicine, University of Washington, Seattle, WA, United States; ^8^ Department of Global Health, University of Washington, Seattle, WA, United States

**Keywords:** bacterial vaginosis, vaginal microbiota, Amsel criteria, Kenya, United States, BVAB1

## Abstract

**Background:**

Bacterial colonization and associations with bacterial vaginosis (BV) signs and symptoms (Amsel criteria) may vary between populations. We assessed relationships between vaginal bacteria and Amsel criteria among two populations.

**Methods:**

Kenyan participants from the placebo arm of the Preventing Vaginal Infections (PVI) trial and participants from a Seattle-based cross-sectional BV study were included. Amsel criteria were recorded at study visits, and the vaginal microbiota was characterized using 16S rRNA gene sequencing. Logistic regression models, accounting for repeat visits as appropriate, were fit to evaluate associations between bacterial relative abundance and each Amsel criterion.

**Results:**

Among 84 PVI participants (496 observations) and 220 Seattle participants, the prevalence of amine odor was 25% and 40%, clue cells 16% and 37%, vaginal discharge 10% and 52%, elevated vaginal pH 69% and 67%, and BV 13% and 44%, respectively. BV-associated bacterium 1 (BVAB1) was positively associated with all Amsel criteria in both populations. *Eggerthella* type 1, *Fannyhessea* (*Atopobium) vaginae*, *Gardnerella* spp., *Sneathia amnii*, and *Sneathia sanguinegens* were positively associated with all Amsel criteria in the Seattle study, and all but discharge in the PVI trial.

**Conclusions:**

Core vaginal bacteria are consistently associated with BV signs and symptoms across two distinct populations of women.

## Introduction

Bacterial vaginosis (BV) is the most common cause of vaginal discharge worldwide, affecting approximately 26% of reproductive-age women (Peebles et al., 2019). BV is a polymicrobial condition characterized by a diverse microbiota of anaerobic and facultative bacteria, including *Gardnerella*, *Prevotella*, *Fannyhessea* (*Atopobium*), and *Sneathia* species. BV etiology remains unclear, in part because vaginal microbiota composition varies between individuals diagnosed with BV and over time within individuals (Srinivasan et al., 2010; Gajer et al., 2012; Srinivasan et al., 2012; Ravel et al., 2013; Bautista et al., 2016; Jung et al., 2017; Muzny et al., 2018; Muzny et al., 2019). Clinical BV diagnosis is based on the presence of at least three of four signs/symptoms termed Amsel criteria: amine odor on addition of potassium hydroxide to vaginal fluid; >20% clue cells on vaginal wet prep; thin, gray, homogeneous vaginal discharge; and elevated vaginal pH >4.5 (Amsel et al., 1983). In research settings, BV is often identified using a Gram stain-based classification system developed by Nugent and Hillier (Nugent et al., 1991). BV symptomatology may vary between women and between global regions, with some studies in sub-Saharan Africa reporting as few as 10% of women with Nugent-BV are symptomatic compared to one third of women with Nugent-BV in North America (Yen et al., 2003; Thoma et al., 2011; Peebles et al., 2019).

It is unclear what drives heterogeneity in BV presentation. A cross-sectional study of women in Seattle, USA identified independent associations between vaginal bacteria and individual Amsel criteria (Srinivasan et al., 2012). These findings suggest BV symptomatology may be driven by the presence or abundance of specific bacteria; however, this has not been assessed in non-US populations. Growing evidence demonstrates that vaginal microbiota composition and associations with clinical outcomes may vary between global populations (Jin et al., 2007; Spear et al., 2008; Lee et al., 2013; Brotman et al., 2014; Benning et al., 2014; Jespers et al., 2014; Balkus et al., 2016; Callahan et al., 2017; McClelland et al., 2018; Bayigga et al., 2019). To investigate the generalizability of findings from the Seattle study, we conducted a comparative analysis of the associations between vaginal bacteria and individual Amsel criteria in a population of Kenyan women enrolled in the Preventing Vaginal Infections (PVI) trial and the Seattle population described above. We performed parallel *de novo* statistical analyses in each study population to facilitate direct comparison of results between the populations.

## Materials and Methods

The PVI trial was a randomized, double-blinded, placebo-controlled trial of periodic presumptive treatment (PPT) for reducing BV and vulvovaginal candidiasis (VVC) (McClelland et al., 2015). The trial and the current study were approved by the Institutional Review Boards of Kenyatta National Hospital, the University of Washington, and the University of Alabama at Birmingham (ClinicalTrails.gov NCT01230814, October 6, 2014). All participants provided written informed consent prior to screening and a second written informed consent if they were eligible and agreed to enroll. The Seattle BV study was cross-sectional and was approved by the Institutional Review Board of the Fred Hutchinson Cancer Research Center (Srinivasan et al., 2012). All participants provided written informed consent prior to enrolling.

### PVI Trial Participants and Study Procedures

PVI trial participants were recruited from four research clinics: one in Mombasa, Kenya, two in Nairobi, Kenya, and one in Birmingham, Alabama (McClelland et al., 2015). The Mombasa clinic and one Nairobi clinic recruited cisgender women who reported transactional sex. The other two clinics recruited cisgender women from the general population. Eligible participants were 18-45 years old, HIV-1 seronegative, and sexually active. Women had to have a vaginal infection at screening: BV (Nugent score ≥7) (Nugent et al., 1991), VVC, and/or *Trichomonas vaginalis* (TV) infection. Women with ≥4 episodes of treatment for symptomatic vaginal infections in the prior year were excluded due to expected need for frequent open-label treatment. At screening, women with symptomatic BV or VVC received open-label treatment, and all women with TV infection received open-label treatment. Women who met enrollment criteria at screening were invited to enroll within 7-28 days. Returning women with no vaginal symptoms were eligible to enroll. During follow-up, participants with symptomatic vulvovaginitis, vaginal discharge, or vaginal itching received open-label treatment. Pelvic speculum examinations were performed and cervicovaginal swabs collected by clinicians at enrollment and follow-up months 2, 4, 6, 8, 10, and 12. At these visits, vaginal saline wet mounts were examined for clue cells, vaginal secretions tested for amine odor, vaginal pH measured using a test strip (EMD Millipore), and vaginal discharge characteristics recorded by clinicians.

Vaginal fluid was collected using a push-off polyester/polyethylene terephthalate swab (FitzCo) for PCR targeting the 16S rRNA gene (V3-V4 hypervariable regions) and Illumina MiSeq sequencing. Approximately 33,000 sequence reads were generated per sample, giving robust detection of minority species. Results were analyzed using a bioinformatics pipeline that overcomes the tendency of sequencing to overestimate species richness (Srinivasan et al., 2012). Sequence data were submitted to the NCBI Short Read Archive (PRJNA638104, **Supplementary Table 1**).

### Seattle Study Participants and Study Procedures

Seattle study participants were recruited from the Public Health Seattle & King County STD Clinic (Srinivasan et al., 2012). Nonpregnant women age 18-50 were eligible, regardless of BV status. Pelvic speculum examinations were performed and cervicovaginal swabs collected by clinicians. Vaginal swabs were used for Gram staining, measuring vaginal pH, saline microscopy, and testing for amine odor. Clinicians recorded vaginal discharge characteristics.

Vaginal fluid was collected using polyurethane foam swabs (Epicentre Biotechnologies) for PCR targeting the 16S rRNA gene (V3-V4 hypervariable regions) and 454 FLX pyrosequencing. Approximately 1,600 sequence reads were generated per sample. Results were analyzed using the same bioinformatics pipeline as above (Srinivasan et al., 2012). Sequence data were submitted to the NCBI Short Read Archive (SRA051298).

### Statistical Analysis

The primary aim of this work was to characterize relationships between vaginal bacteria and Amsel criteria that are qualitatively consistent between the PVI and Seattle populations. Parallel *de novo* statistical analyses were conducted for both populations to enable direct comparison of results between the populations. The current PVI analysis was limited to participants enrolled in Kenya and randomized to placebo because the PPT intervention impacted vaginal bacterial colonization (Balkus et al., 2016). PVI trial observations immediately following open-label treatment were excluded from this analysis. Descriptive statistics were used to summarize participant demographic and behavioral characteristics; Amsel criteria prevalence; and bacterial detection and relative abundance. For each study population, 16S rRNA gene sequencing data were restricted to bacterial taxa whose mean relative abundance was in the top 25% of mean relative abundances for that study and whose prevalence of detection was ≥5% for that study. These filters limited the current analyses to taxa that were commonly detected and that are present at high enough abundances that they may reasonably have a biological impact on the vaginal environment. Amsel criteria (amine odor, clue cells, vaginal discharge, and elevated vaginal pH) were analyzed as individual outcomes and modeled as binary variables (Amsel et al., 1983). Hypothesis tests were considered significant at p<0.05, and a 5% Benjamini-Hochberg false discovery rate was applied to all hypothesis tests. All analyses were conducted separately in each study population, were complete case analyses, and were performed in R (version 3.6.1).

For each study population, taxa of interest for modeling were identified using Analysis of Composition of Microbiomes (ANCOM) (Mandal et al., 2015). ANCOM detects taxa that are differentially abundant between sample types (samples with and without a given Amsel criterion present). ANCOM was specifically designed to be used with compositional 16S rRNA gene sequencing data, as compared to elasticnet regression, which was used in the original Seattle analysis and does not account for compositionality (Fernandes et al., 2014; Mandal et al., 2015). ANCOM was performed separately for each outcome in both study populations.

Bacterial taxa identified as differentially abundant between samples with and without an Amsel criterion present in ANCOM analysis were included in modeling analysis. For each study, four sets of logistic regression models were fit. Each set of models had a single Amsel criterion as the outcome. Within each set, individual models were fit to evaluate associations between the Amsel criterion and each taxon identified as differentially abundant between samples with and without that Amsel criterion. Bacterial relative abundances were modeled using four-level ordinal variables with levels: no detection (reference), tertile 1, tertile 2, and tertile 3. Tertiles of bacterial relative abundance were calculated for each taxon separately in each study population using all samples in which the taxon was detected. Using ordinal exposure variables generates a single odds ratio (OR) for the association between a given taxon and a given Amsel criterion. This OR represents the relative change in odds of the Amsel criterion comparing: samples in relative abundance tertile 1 to samples with no detection; samples in tertile 2 to tertile 1; and samples in tertile 3 to tertile 2. Generalized estimating equations (independent correlation structure) were used for PVI models to account for correlated data due to repeated measures within participants and different numbers of measurements between participants. No exposure variables were lagged in PVI models to allow for cross-sectional interpretation, enabling direct comparison of PVI and Seattle results. All models for both study populations were unadjusted due to the *a priori* hypothesis that no available Seattle study covariates would act as confounders, and to enable direct comparison of PVI and Seattle results.

## Results

Of 234 PVI participants, 221 (94%) returned for at least one follow-up visit and consented to additional testing of stored specimens. One hundred ten (50%) of these participants were randomized to placebo, of whom 84 (76%) were enrolled in Kenya and included in this analysis. Median age was 29 years (interquartile range (IQR): 23.8–33 (**Table 1**). Of 242 Seattle participants, 220 (91%) participants’ 16S rRNA gene sequencing data met previously-described quality measures and were included in this analysis (Srinivasan et al., 2012). Median age was 27 years (IQR: 22-33). Prevalence of BV assessed by Amsel criteria was 13% during PVI follow-up and 44% at the Seattle study visit (Amsel criteria prevalence in **Table 1**). Additional demographic and clinical data are presented in **Table 1**.

**Table 1:** Baseline characteristics and Amsel criteria prevalence in the PVI trial and Seattle study populations.

Characteristic	PVI trial enrollment (N=84)		Seattle study (N=220)	
	N	%	N	%
Race				
American Indian or Alaskan Native	0	0	7	3
Asian	0	0	15	7
Black	84	100	75	34
Mixed race	0	0	12	6
Native Hawaiian or Pacific Islander	0	0	5	2
White	0	0	97	44
Age^a^	29	24 - 33	27	22 - 33
Nugent score				
BV Negative (0-3)	36	43	90	41
Intermediate (4-6)	18	21	13	6
BV Positive (7-10)	30	36	117	53
Clinical factors^b^				
* Chlamydia trachomatis* infection	6	7	–	–
* Trichomonas vaginalis* infection	0	0	15	7
Herpes simplex virus type-2 seropositive	55	66	–	–
Vulvovaginal candidiasis	17	20	17	8
Cervicitis	2	2	–	–
	**PVI trial follow-up (N=496)**		**Seattle study (N=220)** ^c^	
	**N**	**%**	**N**	**%**
Amsel criteria & BV				
Amine odor	124	25	87	40
Clue cells	79	16	81	37
Vaginal discharge	51	10	103	52
Elevated vaginal pH	344	69	145	67
BV assessed by Amsel criteria^d^	66	13	97	44

a Age is reported as median, interquartile range.

b Testing for Chlamydia trachomatis, Herpes simplex virus type-2, and cervicitis was not performed in the Seattle study. No PVI trial participants tested positive for Neisseria gonorrhoeae at enrollment, and testing for N. gonorrhoeae was not performed in the Seattle study.

c Outcomes were not assessed for all participants of the Seattle study. Amine odor, clue cells, and BV were only assessed for 219 participants, vaginal discharge for 200 participants, and vaginal pH for 218 participants. Percentages for these variables used the number of participants for which the outcome was assessed as the denominator.

d BV was defined based on the presence of at least three of four Amsel criteria. PVI, Preventing Vaginal Infections; BV, bacterial vaginosis.

**Figure 1:**
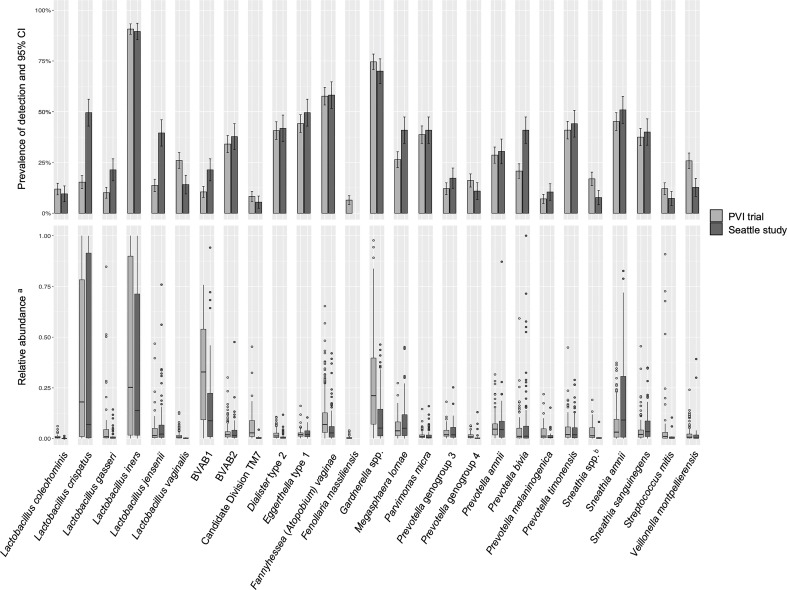
Detection and relative abundance of bacteria included in modeling analysis. All bacteria identified by ANCOM as significantly differentially abundant between samples with and without an Amsel criterion in either study population were included as exposures in modeling analysis and are presented in this figure. ^a^Relative abundances are presented for samples in which a given taxon was detected. ^b^ *Sneathia* spp. includes those not classified at the species level as *S*. *amnii* or *S*. *sanguinegens*, but closely related to these two species. Sequences are not sufficiently different at the V3-V4 region of the 16S rRNA gene to classify at the species level, hence classified at the genus level. PVI, Preventing Vaginal Infections; CI, confidence interval; BVAB, bacterial vaginosis associated bacterium; ANCOM, analysis of composition of microbiomes.

Prevalence of detection and relative abundance of bacterial taxa identified by ANCOM (for any outcome in either population) are summarized in **Figure 1**. *Lactobacillus vaginalis*, *Sneathia* spp. (closely related to *Sneathia amnii* and *Sneathia sanguinegens* but not sufficiently different in the V3-V4 region of the 16S rRNA gene sequences for species-level placement), *Streptococcus mitis*, and *Veillonella montpellierensis* were detected more frequently among PVI samples. *Lactobacillus crispatus*, *Lactobacillus gasseri*, *Lactobacillus jensenii*, BV associated bacterium 1 (BVAB1), *Megasphaera lornae*, and *Prevotella bivia* were detected more frequently among Seattle samples. Among samples in which a given taxon was detected, median relative abundances of *L. crispatus*, *Lactobacillus iners*, BVAB1, Candidate Division TM7, and *Gardnerella* spp. were higher for PVI samples. Median relative abundance of *S. amnii* was higher for Seattle samples.

**Figure 2:**
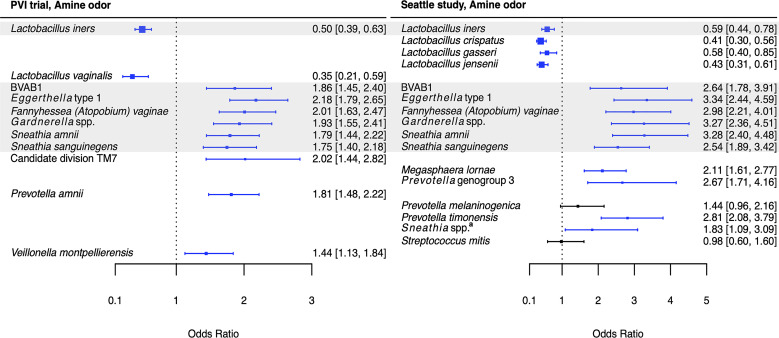
Logistic regression modeling results for the association between vaginal bacteria and amine odor. Gray shading indicates taxa that were modeled in both study populations. Blue box and whiskers indicate associations that were significant following Benjamini-Hochberg multiple comparisons adjustment (5% false discovery rate), and black box and whiskers indicate associations that were not significant following multiple comparisons adjustment. All models were univariable logistic regression models with the Amsel criterion as the outcome and bacterial relative abundance as the exposure. Bacterial relative abundances were modeled as tertiles with the reference level being no detection (four-level ordinal variables with levels: no detection, tertile 1, tertile 2, tertile 3). Generalized estimating equations with an independent correlation structure were used for PVI trial models, and no variables were lagged in PVI models. ^a^ *Sneathia* spp. includes those not classified at the species level as *S*. *amnii* or *S*. *sanguinegens*, but closely related to these two species. Sequences are not sufficiently different at the V3-V4 region of the 16S rRNA gene to classify at the species level, hence classified at the genus level. PVI, Preventing Vaginal Infections; OR, odds ratio; CI, confidence interval; BVAB, bacterial vaginosis associated bacterium.

**Figure 3:**
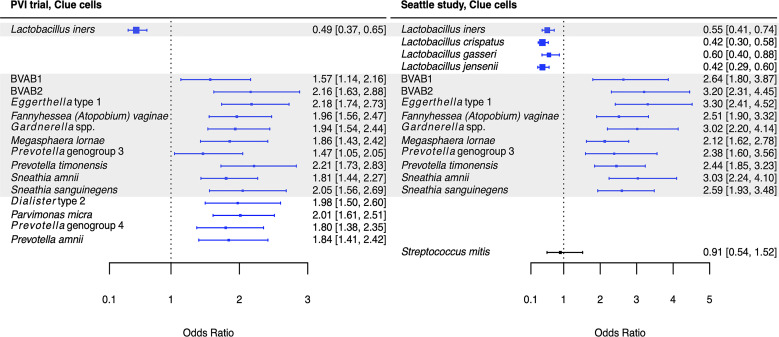
Logistic regression modeling results for the association between vaginal bacteria and clue cells. Gray shading indicates taxa that were modeled in both study populations. Blue box and whiskers indicate associations that were significant following Benjamini-Hochberg multiple comparisons adjustment (5% false discovery rate), and black box and whiskers indicate associations that were not significant following multiple comparisons adjustment. All models were univariable logistic regression models with the Amsel criterion as the outcome and bacterial relative abundance as the exposure. Bacterial relative abundances were modeled as tertiles with the reference level being no detection (four-level ordinal variables with levels: no detection, tertile 1, tertile 2, tertile 3). Generalized estimating equations with an independent correlation structure were used for PVI trial models, and no variables were lagged in PVI models. PVI, Preventing Vaginal Infections; OR, odds ratio; CI, confidence interval; BVAB, bacterial vaginosis associated bacterium.

**Figure 4:**
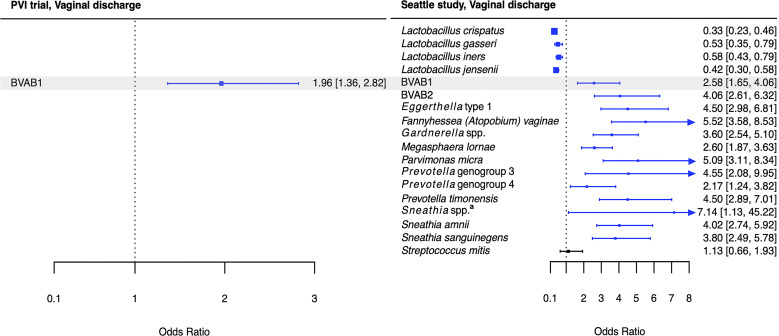
Logistic regression modeling results for the association between vaginal bacteria and vaginal discharge. Gray shading indicates taxa that were modeled in both study populations. Blue box and whiskers indicate associations that were significant following Benjamini-Hochberg multiple comparisons adjustment (5% false discovery rate), and black box and whiskers indicate associations that were not significant following multiple comparisons adjustment. All models were univariable logistic regression models with the Amsel criterion as the outcome and bacterial relative abundance as the exposure. Bacterial relative abundances were modeled as tertiles with the reference level being no detection (four-level ordinal variables with levels: no detection, tertile 1, tertile 2, tertile 3). Generalized estimating equations with an independent correlation structure were used for PVI trial models, and no variables were lagged in PVI models. ^a^ *Sneathia* spp. includes those not classified at the species level as *S*. *amnii* or *S*. *sanguinegens*, but closely related to these two species. Sequences are not sufficiently different at the V3-V4 region of the 16S rRNA gene to classify at the species level, hence classified at the genus level. PVI, Preventing Vaginal Infections; OR, odds ratio; CI, confidence interval; BVAB, bacterial vaginosis associated bacterium.

**Figure 5:**
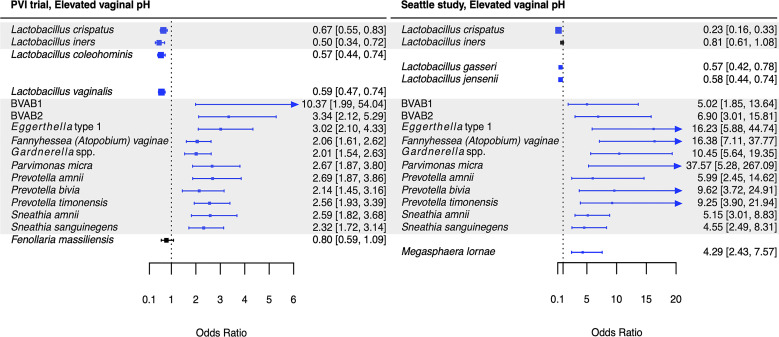
Logistic regression modeling results for the association between vaginal bacteria and elevated vaginal pH. Gray shading indicates taxa that were modeled in both study populations. Blue box and whiskers indicate associations that were significant following Benjamini-Hochberg multiple comparisons adjustment (5% false discovery rate), and black box and whiskers indicate associations that were not significant following multiple comparisons adjustment. All models were univariable logistic regression models with the Amsel criterion as the outcome and bacterial relative abundance as the exposure. Bacterial relative abundances were modeled as tertiles with the reference level being no detection (four-level ordinal variables with levels: no detection, tertile 1, tertile 2, tertile 3). Generalized estimating equations with an independent correlation structure were used for PVI trial models, and no variables were lagged in PVI models. PVI, Preventing Vaginal Infections; OR, odds ratio; CI, confidence interval; BVAB, bacterial vaginosis associated bacterium.

Associations between *Lactobacillus* species and Amsel criteria were largely population-dependent (**Figures 2**–**5**). Higher relative abundance of *L. crispatus* was associated with the absence of all Amsel criteria among Seattle participants, whereas it was only associated with the absence of elevated vaginal pH among PVI participants. Higher relative abundances of *L. gasseri* and *L. jensenii* were associated with the absence of all Amsel criteria among Seattle participants and were not associated with any criteria among PVI participants. Higher *L. iners* relative abundance was associated with the absence of amine odor and clue cells in both populations, the absence of vaginal discharge among Seattle participants, and the absence of elevated vaginal pH among PVI participants.

**Figure 6:**
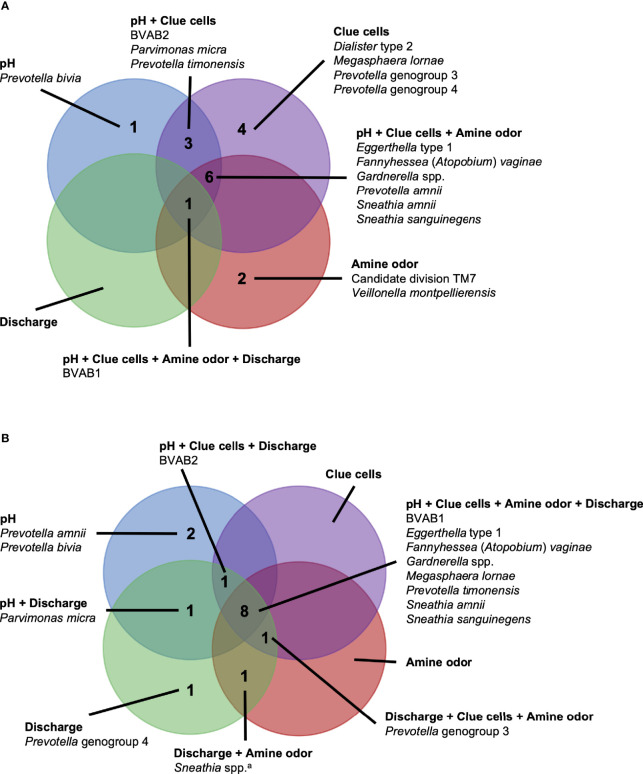
Vaginal bacteria significantly positively associated with Amsel criteria in the PVI trial population and Seattle study population. Vaginal bacteria significantly positively associated with at least one Amsel criterion in **(A)** the PVI trial population and **(B)** the Seattle study population. Results are from logistic regression models fit separately for each study population. In each model, a single Amsel criterion was the outcome, and a single bacterial taxon was the exposure. Bacterial relative abundances were modeled as tertiles with the reference level being no detection (four-level ordinal variables with levels: no detection, tertile 1, tertile 2, tertile 3). Generalized estimating equations with an independent correlation structure were used for PVI trial models, and no variables were lagged in PVI trial models. All models were univariable. All associations represented in the Venn diagrams were significant following Benjamini-Hochberg multiple comparisons adjustment (5% false discovery rate). ^a^ *Sneathia* spp. includes those not classified at the species level as *S*. *amnii* or *S*. *sanguinegens*, but closely related to these two species. Sequences are not sufficiently different at the V3-V4 region of the 16S rRNA gene to classify at the species level, hence classified at the genus level. PVI, Preventing Vaginal Infections; BVAB, bacterial vaginosis associated bacterium.

Several associations between BV-associated taxa and Amsel criteria were consistent between the populations (**Figures 2**–**6**). Higher BVAB1 relative abundance was associated with the presence of all Amsel criteria in both populations. Higher *Eggerthella* type 1, *Fannyhessea* (*Atopobium*) *vaginae*, *Gardnerella* spp., *S. amnii*, and *S. sanguinegens* relative abundances were associated with the presence of all criteria among Seattle participants, and all criteria except discharge among PVI participants. Higher *P. bivia* relative abundance was associated with elevated vaginal pH in both populations. The magnitude of these shared associations was consistently larger among Seattle participants than PVI participants (**Figures 2**–**5**).

For several additional BV-associated taxa, only some associations were shared between the PVI and Seattle populations (**Figures 2**–**6**). Higher *Prevotella timonensis* and BVAB2 relative abundances were associated with the presence of clue cells and elevated vaginal pH in both populations. Both taxa were associated with the presence of discharge among Seattle participants, and *P. timonensis* was associated with the presence of amine odor among Seattle participants. Higher *Parvimonas micra* relative abundance was associated with elevated vaginal pH in both populations. It was also associated with the presence of clue cells among PVI participants and with the presence of discharge among Seattle participants. Higher *Prevotella amnii* relative abundance was associated with elevated vaginal pH in both populations. It was also associated with the presence of amine odor and clue cells among PVI participants. Higher *Prevotella* genogroup 3 and *M. lornae* relative abundances were associated with the presence of clue cells in both populations. Both taxa were associated with the presence of amine odor and discharge among Seattle participants. *M. lornae* was also associated with elevated vaginal pH among Seattle participants.

## Discussion

In this comparative study, a core group of six vaginal bacteria, BVAB1, *Eggerthella* type 1, *F. vaginae*, *Gardnerella* spp., *S. amnii*, and *S. sanguinegens*, were associated with the presence of three to four Amsel criteria in research populations enrolled in Mombasa and Nairobi, Kenya and Seattle, USA. This core group of vaginal bacteria may play a key role in the manifestation of BV signs and symptoms across diverse populations. Although the etiology of BV remains unclear, these results support recent hypotheses that emphasize the formation of a polymicrobial biofilm, possibly initiated by *Gardnerella* and with early inclusion of *F. vaginae* (Swidsinski et al., 2008; Verstraelen and Swidsinski, 2013; Hardy et al., 2015; Jung et al., 2017; Muzny et al., 2018; Muzny et al., 2019). The shared associations between these six bacteria and Amsel criteria may reflect bacterial cooccurrence in the BV biofilm that persists across populations. They may also indicate bacterial interactions within the BV biofilm, which have been documented for *Gardnerella* and *F. vaginae* (Castro et al., 2020).

Consistent with the current Seattle analysis, *Eggerthella* type 1 and *S. amnii* were associated with the presence of all Amsel criteria in the original Seattle analysis published by Srinivasan and colleagues, and *F. vaginae* and *Gardnerella* spp. were associated with three criteria (Srinivasan et al., 2012). However, BVAB1 was only associated with amine odor in the original analysis, and *S. sanguinegens* was not associated with any criteria (complete comparison of original Seattle, current Seattle, and current PVI analyses in **Supplementary Table 2**) (Srinivasan et al., 2012). These and other differences in Seattle analysis results are likely due to differences between elasticnet regression and univariable logistic regression, used in the original and current analyses, respectively (Srinivasan et al., 2012). Elasticnet regression is a predictive modeling approach in which features retained in the final model are those that are most strongly predictive of the outcome, conditional on other features retained in the model (Zou and Hastie, 2005). The lack of association between Amsel criteria and BVAB1 and *S. sanguinegens* in the original analysis reflects that these taxa’s relative abundances did not substantially improve the elasticnet model’s accuracy in predicting Amsel criteria. In the current univariable logistic regression analysis, associations between higher BVAB1 and *S. sanguinegens* relative abundances and the presence of Amsel criteria indicate that these taxa’s relative abundances were significantly higher among samples with Amsel criteria present. The original and current Seattle results are not necessarily inconsistent, and both analytic approaches are hypothesis-generating and require mechanistic studies to understand the role of vaginal bacteria in generating BV signs and symptoms.

Studies investigating vaginal bacterial functions during BV suggest two biologically plausible mechanisms by which this core group of six bacteria may contribute to BV symptomatology. The first potential mechanism is through biogenic amine production. Genomic analyses demonstrated that *Eggerthella* isolates contain genes encoding biogenic amine-producing proteins, and a metabolomics study found that *Eggerthella* type 1, *F. vaginae*, *Gardnerella* spp., *S. amnii*, and *S. sanguinegens* were highly correlated with several biogenic amines, which were in turn associated with BV and individual Amsel criteria (Nelson et al., 2015; Srinivasan et al., 2015). Biogenic amines may contribute to transudation of fluid into the vagina and squamous cell exfoliation, resulting in vaginal discharge and clue cell formation (Sobel, 2000; Yeoman et al., 2013; Srinivasan et al., 2015). They are also involved in amine odor, and production of biogenic amines may consume protons, increasing vaginal pH (Nelson et al., 2015). The second potential mechanism involves production of enzymes that degrade cervicovaginal mucus and epithelium, including sialidases and phospholipases. Studies evaluating the role of extracellular enzymes in vaginal epithelial cell sloughing showed that *Gardnerella* spp. produced sialidases and phospholipases, and high abundances of *F. vaginae* and *Sneathia* were associated with increased sialidase activity during BV (Yeoman et al., 2010; Marconi et al., 2013; Jung et al., 2017). These enzymes may contribute to squamous cell exfoliation and subsequent clue cell formation, and they likely enhance vaginal discharge (Yeoman et al., 2010).

While these potential mechanisms do not appear to involve BVAB1, it is notable that BVAB1 was the only taxon associated with the presence of vaginal discharge in both the PVI and Seattle populations. In addition to BVAB1, 12 other bacteria were associated with vaginal discharge among Seattle participants. The presence of vaginal discharge characteristic of BV was recorded by clinicians in both studies, so these differences are not due to differences in vaginal discharge self-report between the populations. Instead, they can be attributed, at least partially, to the fact that ANCOM only identified BVAB1 as differentially abundant between PVI samples with and without vaginal discharge, making BVAB1 the only bacterial exposure modeled for discharge in the PVI population. Vaginal discharge was only present at 10% of PVI visits, compared to 52% of Seattle visits, and ANCOM may be underpowered to detect taxa that are differentially abundant between PVI samples with and without discharge present. These differences may also indicate that vaginal discharge arises through differing microbial pathways in different populations. Metabolomics evidence again indicates that biogenic amines may contribute to vaginal discharge in the Seattle population. In addition to the core group bacteria, BVAB2, *M. lornae*, *P. micra*, *P. timonensis*, and *Sneathia* spp. were associated with the presence of discharge among Seattle participants. A metabolomics study reported that these taxa correlated strongly with the polyamine cadaverine, which was associated with vaginal discharge (Srinivasan et al., 2015).

Unlike for vaginal discharge, several taxa in addition to the core group bacteria were associated with the presence of clue cells in both the PVI and Seattle populations: BVAB2, *M. lornae*, *P. timonensis*, and *Prevotella* genogroup 3. Prior analysis of the Seattle data also found that BVAB2 and *M. lornae* were associated with clue cells (Srinivasan et al., 2012). Results from the current analyses and prior Seattle analysis are supported by observations from studies evaluating vaginal bacterial functions in BV. Fluorescence *in situ* hybridization targeting BVAB2 showed that BVAB2 can attach to vaginal epithelial cells resulting in similar appearance to clue cells (Fredricks et al., 2005). Other studies investigating vaginal epithelial cell sloughing showed *Prevotella* can produce sialidases and glycosidases, suggesting *Prevotella* may contribute to squamous cell exfoliation and the formation of clue cells (Briselden et al., 1992; Olmsted et al., 2003; Yeoman et al., 2010; Marconi et al., 2013). Likewise, high abundance of *Megasphaera* spp. during BV was associated with high sialidase activity (Marconi et al., 2013). Metabolomics analyses demonstrated that BVAB2, *M. lornae*, and *P. timonensis* were highly correlated with the lipid deoxycarnitine, which was in turn associated with the presence of clue cells (Srinivasan et al., 2015). *Prevotella* are also associated with the polyamine putrescine and contain genes encoding biogenic amine-producing proteins, and polyamines may be involved clue cell formation as discussed above (Sobel, 2000; Yeoman et al., 2013; Nelson et al., 2015). These reports suggest biologically plausible mechanisms that support the epidemiologic associations of BVAB2, *M. lornae*, *P. timonensis*, and *Prevotella* genogroup 3 with the presence of clue cells.

Our results also suggest *Prevotella* may contribute to elevated vaginal pH. In both populations, *P. amnii*, *P. bivia*, *P. timonensis*, BVAB2, and *P. micra* were associated with elevated vaginal pH, in addition to the core group bacteria. Srinivasan and colleagues’ prior analysis of the Seattle data also reported that *P. bivia* was associated with elevated vaginal pH (Srinivasan et al., 2012). Metabolomic and genomic evidence again suggests biogenic amines may underlie these associations. One metabolomics study observed that cadaverine and tyramine, both amines, and N-acetylputrescine, a degradation product of the amine putrescine, were associated with elevated vaginal pH (Srinivasan et al., 2015). These amines were highly correlated with BVAB2 and *P. timonensis*, and cadaverine and N-acetylputrescine with *P. micra* (Srinivasan et al., 2015). A second metabolomics study reported that *Prevotella* are associated with putrescine, and genomic data indicate that various *Prevotella* species contain genes encoding biogenic amine-producing proteins (Yeoman et al., 2013; Nelson et al., 2015). These prior reports and the current analyses’ results are consistent with Nelson and colleagues’ proposed model that biogenic amine production by vaginal bacteria consumes protons, increasing vaginal pH (Nelson et al., 2015).

Several features of the Seattle study, PVI trial, and current analyses are important to consider in interpreting these findings. Although statistical analyses were conducted to enable direct comparison of PVI and Seattle results, technical variation between the studies may influence differences in results. Mean sequencing depth of Seattle samples was 1,620 reads per sample, compared to 33,467 reads per sample for PVI samples as different sequencing platforms (pyrosequencing versus Illumina Mi-Seq, respectively) were used based on availability (Srinivasan et al., 2012). Minority taxa were less likely to be detected in Seattle samples due to lower sequencing depth, and relative abundances may be overestimated in Seattle data (Weiss et al., 2017). Additional study design differences and sources of technical variation between the parent studies may limit our ability to detect associations that are consistent between the two populations, which is why we focus on associations that are consistent between the populations. Moreover, that we were able to detect several shared associations between the populations despite this variation strengthens the evidence for these relationships. Finally, 16S rRNA gene sequencing facilitates taxonomic identification at the genus or species levels for most taxa. For example, 16S rRNA gene sequencing does not differentiate between the recently identified *Gardnerella* species (Vaneechoutte et al., 2019), which may have different relationships with BV signs and symptoms. We were unable to examine heterogeneity between *Gardnerella* species because both original studies used 16S rRNA gene sequencing and therefore classified *Gardnerella* at the genus level.

This comparison study indicates that a core group of vaginal bacteria may contribute to BV symptomatology across populations, and that additional vaginal bacteria may play a role in the manifestation of specific BV signs and symptoms. While these relationships should be investigated in additional populations, these findings help contextualize heterogeneity in BV symptomatology between women and across global regions. These findings are also consistent with growing evidence regarding the role of biogenic amines and extracellular enzymes in BV etiology and symptom manifestation. Taken together, this work and prior reports suggest that bacteria highlighted here may consistently contribute to BV symptomatology in various populations, despite differences in overall vaginal microbiota composition.

## Data Availability Statement

Publicly available datasets were analyzed in this study. This data can be found here: The Seattle study datasets analyzed for this study can be found in the NCBI Short Read Archive (SRA051298, https://www.ncbi.nlm.nih.gov/sra/?term=SRA051298) and the supplementary materials of the original Seattle study publication (Srinivasan et al. PLoS ONE. 2012. DOI: 10.1371/journal.pone.0037818, https://journals.plos.org/plosone/article?id=10.1371/journal.pone.0037818, https://doi.org/10.1371/journal.pone.0037818.s006, https://doi.org/10.1371/journal.pone.0037818.s007). The PVI trial datasets analyzed for this study can be found in the NCBI Short Read Archive (PRJNA638104, https://www.ncbi.nlm.nih.gov/bioproject/PRJNA638104/) and this publication’s **Supplementary Material**.

## Ethics Statement

The studies involving human participants were reviewed and approved by the Institutional Review Boards of Kenyatta National Hospital, the University of Washington, and the University of Alabama at Birmingham (PVI trial, ClinicalTrails.gov NCT01230814, October 6, 2014); and the Institutional Review Board of the Fred Hutchinson Cancer Research Center (Seattle BV study). The patients/participants provided their written informed consent to participate in this study.

## Author Contributions

TF, DF, and SS contributed to Seattle study design, implementation, and data collection. JB and RM contributed to PVI trial design. JB, OA, JK, VM, and RM contributed to PVI trial implementation and data collection. NH, TF, DF, and SS contributed to PVI trial and Seattle study microbiota characterization. NH, TF, DF, and SS contributed to the original Seattle study statistical analysis. KC, JB, and SS contributed to the current PVI trial and Seattle study statistical analyses. KC wrote the first manuscript draft and led manuscript writing. JB and SS contributed substantially to early manuscript revisions. KC, JB, OA, JK, NH, TF, VM, DF, RM, and SS contributed to, reviewed, and approved the final manuscript.

## Funding

This work was supported by the National Institute of Allergy and Infectious Diseases of the National Institutes of Health [grant number R01 AI061628 to the Seattle study, grant number R01 AI099106 to the Preventing Vaginal Infections trial microbiota characterization, and contract number HHSN266200400073C through the Sexually Transmitted Infections Clinical Trials Group grant number STI CTG 09-0070 to the Preventing Vaginal Infections trial]; the National Human Genome Research Institute of the National Institutes of Health [grant number R01 HG005966-01 to the Seattle study]; and the National Institutes of Health [grant number K24 HD882 to R.S.M. and grant number T32 AI07140 to KC]. The Preventing Vaginal Infections trial received funding (donated study product) from Embil Pharmaceutical Company. Embil Pharmaceutical Company was not involved in the study design, collection, analysis, interpretation of data, the writing of this article or the decision to submit it for publication.

## Conflict of Interest

JB has received honoraria from BD for consulting. RM has received research funding, paid to the University of Washington, from Hologic Corporation and has received honoraria for consulting from Lupin Pharmaceuticals. SS has received consulting honoraria from Lupin Pharmaceuticals. DF and TF received a royalty from BD.

The remaining authors declare that the research was conducted in the absence of any commercial or financial relationships that could be construed as a potential conflict of interest.

The Preventing Vaginal Infections trial received funding (donated study product) from Embil Pharmaceutical Company. Embil Pharmaceutical Company was not involved in the study design, collection, analysis, interpretation of data, the writing of this article or the decision to submit it for publication.

## Publisher’s Note

All claims expressed in this article are solely those of the authors and do not necessarily represent those of their affiliated organizations, or those of the publisher, the editors and the reviewers. Any product that may be evaluated in this article, or claim that may be made by its manufacturer, is not guaranteed or endorsed by the publisher.
