# Supplementary material for: Associations Between Vaginal Bacteria and Bacterial Vaginosis Signs and Symptoms: A Comparative Study of Kenyan and American Women
Source: Front Cell Infect Microbiol. 2022 Mar 4;12:801770. doi: 10.3389/fcimb.2022.801770 (PMC8931342; doi:10.3389/fcimb.2022.801770)
Supplement: Supplementary file 2 [file Table_1.docx]

Supplementary Material

# Supplementary Tables

**Supplementary Table 1. Amsel criteria and covariate metadata for PVI trial Sequence Read Archive submission.**

PVI trial 16S rRNA gene sequencing data were submitted to the NCBI Short Read Archive (Accession: PRJNA638104). Abbreviations: PVI, Preventing Vaginal Infections.

**Supplementary Table 2. Comparison of bacterial detection, modeling, and associations between original Seattle analysis, current Seattle analysis, current PVI analysis.**

All unshaded (white background) taxa were modeled in all three analyses (N=16). Orange shading indicates taxa that were modeled in the original and current Seattle analyses, but not in the current PVI analysis (N=5). Purple/gray shading indicates taxa that were modeled in the original Seattle analysis and current PVI analysis, but not in the current Seattle analysis (N=5). Yellow shading indicates taxa that were modeled in the current PVI analysis, but not in the original or current Seattle analyses (N=1). Green shading indicates taxa that were modeled in the original Seattle analysis, but not in the current Seattle analysis or the current PVI analysis (N=5). ^a^Includes all taxa associated with >1 Amsel criterion in the original Seattle analysis and all taxa significantly associated with >1 Amsel criterion in the current Seattle and PVI analyses following multiple comparisons adjustment (5% false discovery rate). ^b^The original Seattle analysis was reported in Srinivasan et al. PloS One 2012;7(6):e37818. ^c^Detected by 16S rRNA gene sequencing in >1 sample. ^d^For the original Seattle analysis, all detected taxa were modeled using elasticnet regression. For the current Seattle and PVI analyses, bacteria meeting the RA cutoff were selected for modeling if they were identified by ANCOM as being significantly differentially abundant between samples with and without an Amsel criterion present. ^e^Amsel criteria include: amine odor on addition of potassium hydroxide to vaginal fluid; >20% clue cells on vaginal wet prep; thin, gray, homogeneous vaginal discharge; and vaginal pH >4.5. ^f^The current Seattle and PVI analyses were restricted to taxa whose mean relative abundance among all samples for a given cohort was among the top 25% of mean relative abundances for the cohort, and whose prevalence of detection in the cohort was >5%. ^g^Reported as *Gardnerella vaginalis* in the original Seattle study publication. ^h^Formerly *Megasphaera* type 1. ^i^Reported as *Leptotrichia amnionii* in the original Seattle study publication. ^j^*Sneathia* spp. include those not classified at the species level as *Sneathia* *amnii* or *Sneathia* *sanguinegens,* but closely related to these two species. Sequences are not sufficiently different at the V3-V4 region of the 16S rRNA gene to classify at the species level, hence classified at the genus level. ^k^*Fenollaria massiliensis* was described in 2017. Seattle 16S rRNA gene sequencing data were generated prior to 2017. No *Fenollaria* species were detected in the Seattle cohort. Abbreviations: PVI, Preventing Vaginal Infections; RA, relative abundance; BVAB, bacterial vaginosis associated bacterium; ANCOM, analysis of composition of microbiomes.
